# A quality improvement project to enhance the knowledge, skills, and attitude of healthcare workers regarding the use of defibrillators

**DOI:** 10.1097/MS9.0000000000002417

**Published:** 2024-08-02

**Authors:** Fahad Gul, Seemab Abid, Shafia Khalid, Shahrukh Khalid, Iram Shad, Samar Saleem, Sardar Noman Qayyum, Samim Noori

**Affiliations:** a Department of Medicine, Holy Family Hospital; b Department of Medicine, Benazir Bhutto Hospital; c Department of Medicine, Rawalpindi Teaching Hospital, Rawalpindi; d Department of Medicine, Bacha Khan Medical College, Mardan, Pakistan; e Nangarhar University, Faculty of Medicine, Nangarhar, Afghanistan

**Keywords:** defibrillation, defibrillator, knowledge, skills, healthcare workers, emergency department, clinical audit

## Abstract

**Introduction:**

Defibrillation is a critical intervention in managing cardiac emergencies, yet healthcare workers (HCWs) preparation for utilizing defibrillators remains inadequate, particularly in low and middle-income countries. This quality improvement project aimed to assess and enhance HCWs’ knowledge, skills, and attitudes toward defibrillator use in the emergency department (ED) through a 1-h defibrillator workshop.

**Methodology:**

An observational clinical audit was conducted within the ED of a tertiary care hospital. Pre- and post-workshop data were collected from the participants using structured questionnaires for demographics, knowledge assessment (20 multiple-choice questions), skills assessment (10-step checklist), and attitude evaluation (Likert-scale statements). The workshop included theoretical instruction and hands-on practice, with a post-workshop assessment conducted one week later. Data analysis employed descriptive statistics and paired *t*-tests, while ethical considerations ensured confidentiality and consent.

**Results:**

The study included 38 participants, demonstrating significant gaps in defibrillator knowledge, skills, and attitudes pre-workshop. Post-workshop assessments revealed a marked improvement in knowledge scores (*P*<0.05), attitudes (*P*<0.05), and practical skills (*P*<0.05). Participants’ confidence and preparation for managing cardiac emergencies notably increased, indicating the workshop’s efficacy in addressing the identified deficiencies.

**Conclusion:**

The 1-h defibrillator workshop effectively enhanced HCWs’ competence and readiness to utilize ED defibrillators. The observed improvements underscore the importance of targeted educational interventions in bridging knowledge gaps and fostering proactive attitudes toward emergency management. Regular training sessions should be conducted to sustain these enhancements and improve patient outcomes in the ED.

## Introduction

Highlights

- The pre-workshop assessments revealed concerning gaps in the knowledge, skills and attitudes of healthcare workers regarding defibrillators use in the emergency department.
- The implementation of a one-hour defibrillator workshop proved highly effective in enhancing healthcare workers’ knowledge, practical skills and attitudes towards defibrillation.
- The post-workshop assessments demonstrated significant improvements in knowledge scores (mean score increased from 12 to 18 out of 20), attitude scores (mean scores increased from 30 to 42 out of 50), and the practical skills scores (mean score increased from 6 to 9 out of 10).
- The study also highlighted the importance of hands-on training and regular skills reinforcement in maintaining competence in defibrillation procedures.
- The findings emphasize the need for incorporating focused educational interventions, such as defibrillator workshops, into ongoing professional development programs for emergency department personnel.

Defibrillation is a life-saving intervention that delivers an electric shock to the heart to restore its normal rhythm in cases of cardiac arrest^1^. It is one of the essential components of cardiopulmonary resuscitation (CPR) and advanced cardiac life support (ACLS)^2^. Defibrillators can deliver defibrillation shocks manually or automatically, depending on the type and setting^3^. In the emergency department (ED), defibrillators with full monitors are commonly used to monitor the patient’s vital signs and provide manual or synchronized shocks as needed^4^. Healthcare workers (HCWs) who work in the ED should be competent and confident in using defibrillators, as they may encounter patients who require urgent defibrillation. Several studies have reported that HCWs’ knowledge and skills in using defibrillators are inadequate or suboptimal, especially in low- and middle-income countries^5–7^. This may lead to delays, errors, or complications in defibrillation, which can adversely affect the patient’s survival and outcome^8^. Therefore, it is imperative to evaluate and enhance healthcare workers’ knowledge and skills in utilizing defibrillators, along with addressing their attitudes and potential barriers to the defibrillation process^9^. This can be achieved by conducting a clinical audit, a systematic method of measuring and improving the quality of care against predefined standards or criteria^10^.

This study aims to assess doctors’ knowledge and skills in using defibrillators in the ED. The specific objectives are:

- To measure doctors’ knowledge, skills, and attitudes in using defibrillators before and after an intervention.
- To evaluate the effectiveness of an intervention (e.g., a defibrillator workshop) in improving doctors knowledge, skills and attitude in using defibrillators.

## Methodology

This observational clinical audit was conducted within the ED of a tertiary care hospital over the time period of 3 weeks, involving all the doctors working in the day-night shift rotation within the ED of the hospital. This clinical audit has been conducted following The Revised Standards for Quality Improvement Reporting Excellence (SQUIRE 2.0) guidelines^11^ and a SQUIRE checklist, Supplemental Digital Content 1, http://links.lww.com/MS9/A576 has also been added.

The clinical audit aimed to evaluate doctors’ baseline knowledge, skills, and attitudes in using defibrillators prior to an educational intervention, and determine the effectiveness of the intervention in improving doctors’ knowledge, skills, and attitudes related to defibrillator use through pre- and post-intervention assessment.

A 2.5-h workshop was designed and implemented as an educational intervention to improve healthcare professionals’ knowledge, skills, and attitudes regarding the utilization of defibrillators in emergency settings. The workshop commenced with a 30-min introductory lecture that provided an overview of cardiac arrest management, the critical role of defibrillators, and the significance of timely defibrillation. Subsequently, a 20-min video was played, demonstrating the proper use of defibrillators, followed by a question-and-answer session.

Participants were then divided into small groups for a 60-min hands-on practice session with defibrillators and simulation mannequins. This practical session was supervised by instructors, enabling participants to apply their acquired knowledge and refine their skills. Furthermore, a 20-min interactive discussion session was conducted, facilitating a dialogue on addressing challenges and best practices associated with defibrillators use in real-world settings. Additionally, instructional videos demonstrating defibrillator use were shared with participants via WhatsApp, reinforcing the learning objectives.

Non-random sampling technique was used for the recruitment of participants. Data were collected pre- and post-workshop through the self-administered structured questionnaires with four sections: demographics and professional information, knowledge assessment (20 multiple-choice questions), skills assessment (10-step checklist), and attitude assessment (10 Likert-scale statements). The questionnaire underwent pilot testing with a sample size of 10 participants before its application in this study.

Data analysis involved descriptive statistics and paired *t*-tests. Ethical considerations included administrative permission, informed consent, and ensuring participant confidentiality, anonymity, privacy, and the right to withdraw. The manuscript followed STROCCS guidelines for reporting the study findings^12^ and a STROCCS checklist has been added in supplementary files, Supplemental Digital Content 2, http://links.lww.com/MS9/A577, Supplemental Digital Content 3, http://links.lww.com/MS9/A578.

## Results

**Figure 1:**
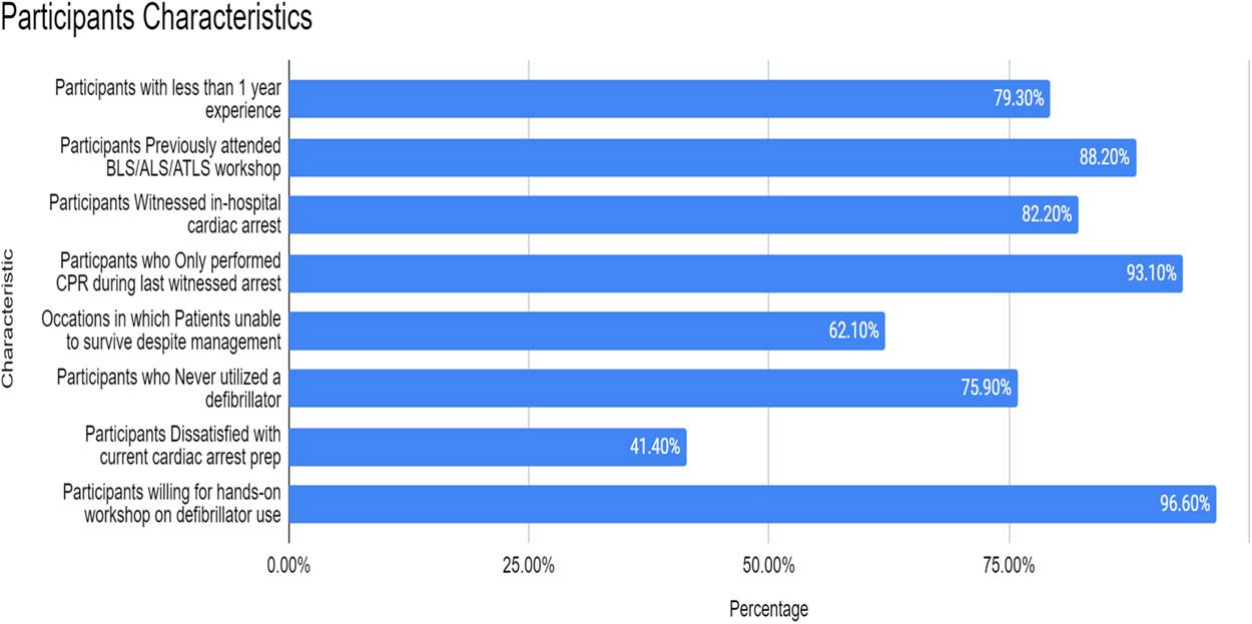
The characteristics of the study participants. CPR, cardiopulmonary resuscitation.

Thirty-eight doctors, with a mean age of 24 ± 3 years, participated in the pre-workshop evaluation. Subsequently, 30 participants attended the one-hour defibrillator workshop, while 28 participated in the post-workshop evaluation. Figure 1 summarizes the characteristics of participants. Most participants (79.3%) had less than one year of experience in the emergency department. Furthermore, 88.2% of the participants had previously attended BLS/ALS/ATLS or any cardiac arrest management workshop. Moreover, a significant proportion of participants (82.2%) reported witnessing an in-hospital cardiac arrest, with 93.1% of these participants having only performed CPR during the last witnessed cardiac arrest.

Notably, a considerable percentage (62.1%) of the participants indicated that patients were unable to survive despite their management during the last cardiac arrest they had encountered. Surprisingly, a significant proportion (75.9%) of participants had never utilized a defibrillator. Furthermore, 41.4% of the participants expressed dissatisfaction with their current preparation for cardiac arrest management, while an overwhelming majority (96.6%) emphasized the necessity of a hands-on workshop on defibrillator use. Figure 1 summarizes the characteristics of the study participants.

### Knowledge assessment

**Figure 2:**
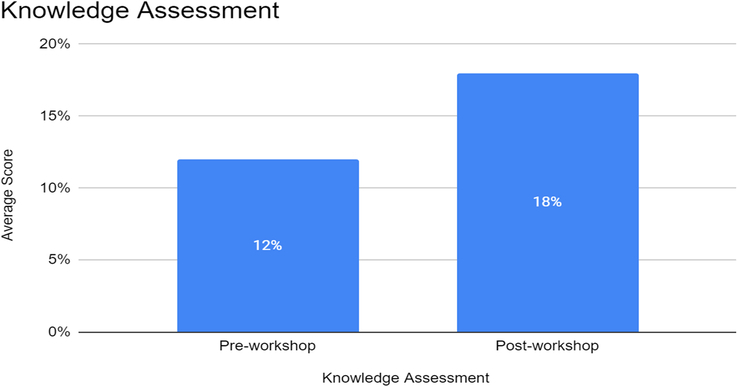
Knowledge assessment of participants pre- and post-workshop.

The pre-workshop knowledge assessment revealed that participants scored an average of 12 out of 20 points (Mean ± SD). Following the workshop, post-workshop knowledge assessments demonstrated a significant improvement in knowledge, with participants achieving an average score of 18 out of 20 points (Mean ± SD). This observed increase in knowledge scores reflects a substantial enhancement in participants’ understanding of defibrillation procedures. The paired *t*-test conducted on the knowledge scores indicated a statistically significant difference (*P*<0.05) between pre- and post-workshop scores. Figure 2 summarizes the study findings related to knowledge assessment pre- and post-workshop.

### Attitude assessment

**Figure 3:**
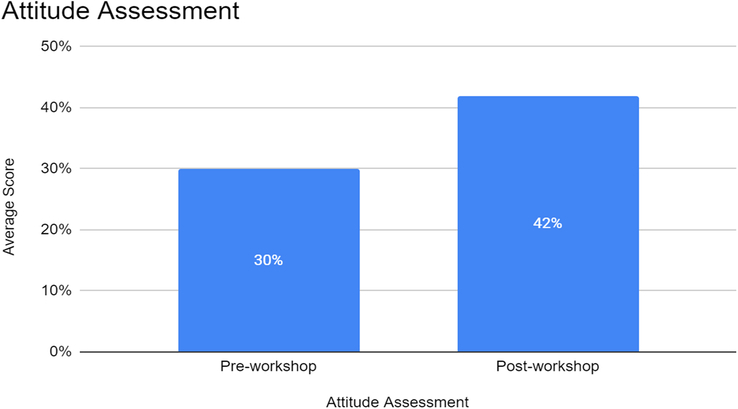
Attitude assessment of participants pre- and post-workshop.

Prior to the workshop, participants’ attitudes towards defibrillation were assessed using a Likert-scale questionnaire, with a maximum attainable score of 50 points. The pre-workshop attitude assessment yielded an average score of 30 (Mean ± SD). After completing the workshop, post-workshop attitude assessments showed a noteworthy improvement in attitudes, with participants scoring an average of 42 points (Mean ± SD). This positive shift in attitude scores indicates an increased confidence and positive disposition towards defibrillation. The paired *t*-test conducted on attitude scores indicated a statistically significant difference (*P*<0.05) between pre- and post-workshop scores. Figure 3 summarized the attitude assessment of the study participants pre- and post-intervention.

### Skills assessment

**Figure 4:**
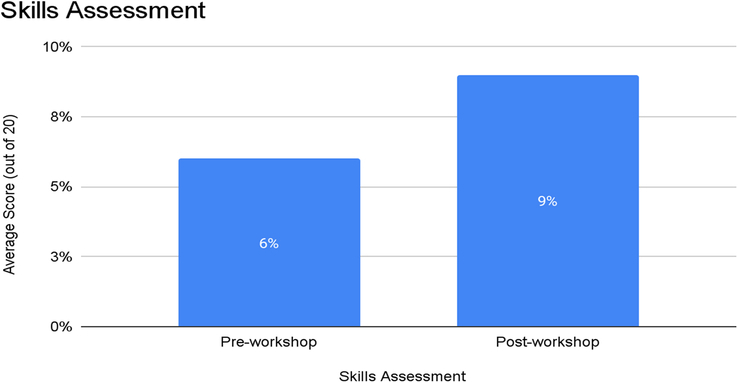
Skill assessment of the participants pre- and post-workshop.

Participants’ skills in using the defibrillator with a full monitor were evaluated through a 10-step checklist during the pre-workshop assessment. The average pre-workshop skills score was 6 out of a possible 10 points (Mean ± SD). Following the workshop, post-workshop skills assessments demonstrated a substantial enhancement in practical skills, with participants achieving an average score of 9 out of 10 points (Mean ± SD). This notable improvement in skills scores indicates a significant advancement in participants’ ability to execute defibrillation procedures correctly. The paired *t*-test conducted on skills scores indicated a statistically significant difference (*P*<0.05) between pre- and post-workshop scores. Figure 4 summarizes the skill assessment of the participants pre- and post-workshop.

### Effectiveness of the workshop

The results clearly demonstrate the effectiveness of the one-hour defibrillator workshop. The significant increase in knowledge, positive shift in attitudes, and improved practical skills post-workshop highlight the positive impact of the intervention. The workshop not only enhanced participants’ theoretical understanding of defibrillation but also provided them with the hands-on experience necessary to perform defibrillation procedures effectively. These findings underscore the importance of targeted educational interventions in improving healthcare workers’ competence and readiness to respond to cardiac emergencies in the ED. The observed improvements in knowledge, attitude, and skills collectively indicate that the workshop effectively addressed the study objectives and contributed to enhancing the participants’ overall preparedness for cardiac arrest management.

## Discussion

The findings of this study highlight the significant impact of a focused educational intervention on improving house officers’ knowledge, attitudes, and skills in using defibrillators for cardiac arrest management in the emergency department. The pre-workshop assessments revealed concerning gaps in these crucial areas, underscoring the need for targeted training initiatives.

The substantial improvement in knowledge scores post-workshop aligns with previous studies that have demonstrated the effectiveness of educational interventions in enhancing healthcare workers’ knowledge related to defibrillation and cardiac arrest management^9,13^. The low pre-workshop knowledge scores, coupled with the high proportion of participants who had attended previous BLS/ALS/ATLS or cardiac arrest management workshops, suggest that knowledge retention may be a challenge without regular reinforcement and hands-on practice^14^.

The positive shift in attitudes towards defibrillation after the workshop is a notable achievement. Favorable attitudes are crucial for ensuring prompt and effective defibrillation, as hesitation or apprehension can lead to delays that compromise patient outcomes^15^. The pre-workshop attitude scores, indicating a relatively neutral stance, highlight the importance of addressing attitudinal barriers and instilling confidence in healthcare workers through targeted training^16^.

The significant improvement in practical skills post-workshop underscores the importance of hands-on training in defibrillation procedures. Theoretical knowledge alone is insufficient; healthcare workers must have the opportunity to practice and develop proficiency in using defibrillators^17^. The low pre-workshop skills scores, despite participants’ prior exposure to cardiac arrest management workshops, suggest a need for regular skills reinforcement and assessment^18^.

The substantial improvements across knowledge, attitudes, and skills demonstrate the effectiveness of the 1-h defibrillator workshop. This finding aligns with previous studies highlighting the benefits of focused, hands-on training interventions for enhancing healthcare workers’ competence in defibrillation and cardiac arrest management^19,20^. The workshop’s positive impact emphasizes the importance of incorporating such targeted educational initiatives into ongoing professional development programs for emergency department personnel.

### Limitations and future outlook

While the study offers valuable insights into improving healthcare professionals’ knowledge, skills and attitudes towards defibrillator use in emergency settings, it is essential to acknowledge certain limitations. The study participants included only doctors working in the emergency department, excluding nurses and other paramedical staff who also play crucial roles in emergency settings as a result the findings may not be generalizable healthcare professionals other than doctors.

Moreover, the non-random sampling technique and small sample size from a single institute may also limit the generalizability of the study findings. The study assessed the efficacy of the workshops only one week after the intervention, which may not have provided insights into long-term retention of knowledge, skills, and attitudes. Additionally, the reliance on self-administered questionnaires for data collection may have introduced social desirability bias and recall bias, and the study did not account for potential confounding factors, such as participant’s prior training experiences, which could have influenced their baseline knowledge, attitudes, and skills.

To address these limitations, multi-center longitudinal studies with larger, randomly selected sample of diverse healthcare professionals, including nurses and other paramedical staff, are required to improve generalizability and evaluate long-term impacts of the workshop on knowledge, skills and attitudes of healthcare workers. These studies should incorporate objective assessments by independent evaluators, investigate impacts on clinical outcomes and patient safety metrics, and take into account potential confounding factors such as prior training. Future research can address these aspects and provide a comprehensive understanding of effective strategies to enhance emergency care, optimizing patient outcomes.

## Conclusion

The implementation of a focused one-hour defibrillator workshop proved highly effective in enhancing house officers’ knowledge, attitudes, and practical skills related to defibrillation in the emergency department. The significant improvements observed across all three domains highlight the importance of targeted educational interventions in addressing gaps and ensuring healthcare workers’ readiness to respond to cardiac emergencies effectively.

The findings underscore the need for regular, hands-on training opportunities and skills reinforcement to maintain competence in defibrillation procedures. Incorporating such workshops into ongoing professional development programs can contribute to improved patient outcomes by ensuring that emergency department personnel are well-equipped to respond promptly and confidently in life-threatening cardiac situations.

Future research could explore the long-term retention of knowledge, attitudes, and skills acquired through such interventions, as well as investigate the impact of periodic refresher training on maintaining competence levels. Additionally, expanding the study to multiple healthcare settings and diverse healthcare worker populations could enhance the generalizability of the findings.

## Ethical approval

Ethical approval for the audit was obtained from the respective Emergency Department.

## Consent

All participants provided informed consent before participating in the study. The confidentiality of the participants’ information was strictly maintained throughout the study, and the data were used only for research purposes. Any identifying information was excluded from the analysis and reporting of the findings to ensure the anonymity of the participants.

## Source of funding

Not applicable.

## Author contribution

F.G., S.A., and S.K. collectively conceived the study, designed its framework, contributed to the write-up, implemented the audit, critically reviewed the content, and approved the final version. F.G. and S.A. also presented the audit findings at the departmental meeting in Holy Family Hospital, Rawalpindi, Pakistan. S.K., I.S., and S.S. contributed to the conceptualization, data collection, manuscript editing and approved the final version. S.N.Q. and S.N. contributed to the write-up, critically reviewed the content, did manuscript edit and approved the final version. Final draft: All authors approved the final manuscript.

## Conflicts of interest disclosure

All authors declare no conflict of interest.

## Research registration unique identifying number (UIN)

Not applicable.

## Guarantor

Sardar Noman Qayyum.

## Data availability statement

The data used to support the findings of this study are available from the corresponding author upon request. Access to the data will be provided in accordance with the institutional regulations and policies governing data sharing and protection.

## Provenance and peer review

Not commissioned and externally peer-reviewed

## Supplementary Material
