# Supplementary material for: A quality improvement project to enhance the knowledge, skills, and attitude of healthcare workers regarding the use of defibrillators
Source: Ann Med Surg (Lond). 2024 Aug 2;86(9):5206–10. doi: 10.1097/MS9.0000000000002417 (PMC11374289; doi:10.1097/MS9.0000000000002417)
Supplement: Supplementary file 3 [file ms9-86-5206-s003.docx]

**Questionnaire**

This questionnaire is designed to collect some basic information about you, such as your age, gender, years of experience, and cardiac arrest management experience. Please answer each question honestly and accurately. Your personal information will be kept confidential and will only be used for the purpose of this study.

1. Age ………….

2. Gender

A) Male

B) Female

5. How many years of experience do you have in working in the emergency department?

……………..

6. Have you attended any BLS/ ALS / ATLS or Cardiac arrest management workshop before ?

Yes

No

7. Have you used a defibrillator before ?

Yes

No

8. Do you have personal experience on cardiac arrest management?

Yes

No

9. Do you know that a defibrillator is available in your ward and emergency ?

Yes

No

10. Are you able to use a defibrillator ?

Yes

No

11 .Have you ever attended an in-hospital cardiac arrest? (If yes, how many ?)

Yes

No

12. How did you take part in the cardiac management in the last cardiac arrest you witnessed?

Did CPR

Used a Defibrillator

Didn't do anything

Other ……

12. What was the outcome of the last cardiac arrest that you witnessed?

Survived

Died

13. How can you judge your preparation on cardiac arrest management?

Good

Sufficient

Not Sufficient

14. Do you think there is a need for a hands-on workshop on a defibrillator use?

Yes

No

**Knowledge Questionnaire**

This questionnaire is designed to measure your knowledge about defibrillation, which is the delivery of an electric shock to the heart to restore its normal rhythm in cases of cardiac arrest. Please read each question carefully and choose the best answer from the four options given. There is only one correct answer for each question. Please mark your answer by circling the letter that corresponds to your choice.

1. What is the most common cause of cardiac arrest in adults?

A) Asphyxia

B) Trauma

C) Ventricular fibrillation

D) Myocardial infarction

2. What is the main purpose of defibrillation?

A) To stop the heart from beating

B) To restart the heart after it has stopped

C) To correct an abnormal heart rhythm

D) To increase the blood pressure and oxygen level

3. What are the two types of defibrillators that are commonly used in the emergency department?

A) Manual and automated

B) Monophasic and biphasic

C) Internal and external

D) Manual and synchronized

4. What are the advantages of using a defibrillator with a full monitor?

A) It can display the patient's electrocardiogram (ECG), blood pressure, oxygen saturation, and other vital signs

B) It can adjust the energy level, mode, and other settings of the shock according to the patient's condition

C) It can record and store the data and events related to the defibrillation process

D) All of the above

5. What are the indications for defibrillation?

A) Pulseless ventricular tachycardia (VT)

B) Ventricular fibrillation (VF)

C) Asystole (flatline)

D) A and B only

6. What are the contraindications for defibrillation?

A) Fine ventricular fibrillation (FVF)

B) Pulseless electrical activity (PEA)

C) Supraventricular tachycardia (SVT)

D) B and C only

7. What are the possible complications of defibrillation?

A) Skin burns

B) Cardiac arrhythmias

C) Myocardial damage

D) All of the above

8. What is the recommended energy level for defibrillation in adults?

A) 50 joules

B) 100 joules

C) 200 joules

D) 360 joules

9. What is the difference between manual and synchronized mode in defibrillation?

A) Manual mode delivers a shock whenever the user presses the shock button, while synchronized mode delivers a shock only when it detects a QRS complex on the ECG

B) Manual mode delivers a shock only when it detects a QRS complex on the ECG, while synchronized mode delivers a shock whenever the user presses the shock button

C) Manual mode delivers a shock with a fixed energy level, while synchronized mode delivers a shock with a variable energy level depending on the patient's impedance

D) Manual mode delivers a shock with a variable energy level depending on the patient's impedance, while synchronized mode delivers a shock with a fixed energy level

10. When should synchronized mode be used in defibrillation?

A) When the patient has pulseless VT or VF

B) When the patient has unstable VT or SVT with a pulse

C) When the patient has asystole or PEA

D) When the patient has atrial fibrillation or flutter

11. What are the steps to perform defibrillation using a defibrillator with full monitor?

A) Turn on the device, select manual or synchronized mode, attach electrodes to chest, check ECG, select energy level, charge device, clear area, deliver shock, check rhythm, repeat if needed

B) Turn on device, attach electrodes to chest, select manual or synchronized mode, check ECG, select energy level, charge device, clear area, deliver shock, check rhythm, repeat if needed

C) Turn on device, attach electrodes to chest, check ECG, select manual or synchronized mode, select energy level, charge device, clear area, deliver shock, check rhythm, repeat if needed

D) Turn on device, check ECG, attach electrodes to chest, select manual or synchronized mode, select energy level, charge device, clear area, deliver shock, check rhythm, repeat if needed

12. Where should the electrodes be placed on the chest for defibrillation?

A) Right upper chest and left lower chest

B) Left upper chest and right lower chest

C) Right upper chest and left upper chest

D) Left lower chest and right lower chest

13. How should the electrodes be attached to the chest for defibrillation?

A) Peel off backing paper and apply firmly to skin

B) Apply conductive gel or paste and press firmly to skin

C) Wet electrodes with saline or water and press firmly to skin

D) Any of the above

14. How should the ECG be checked before defibrillation?

A) Look at the monitor screen and identify the rhythm

B) Feel for a pulse and confirm the rhythm

C) Listen to the device's voice prompts and follow the instructions

D) A and B only

15. How should the energy level be selected for defibrillation?

A) Use the default energy level set by the device

B) Use the lowest energy level that is effective

C) Use the highest energy level that is safe

D) Use the energy level recommended by the guidelines

16. How should the device be charged before defibrillation?

A) Press and hold the charge button until the device is fully charged

B) Press and release the charge button and wait for the device to be fully charged

C) Press the charge button twice and wait for the device to be fully charged

D) Any of the above

17. How should the area be cleared before defibrillation?

A) Shout "clear" and make sure no one is touching the patient or the bed

B) Shout "stand clear" and make sure no one is touching the patient or the bed

C) Shout "I'm clear, you're clear, we're all clear" and make sure no one is touching the patient or the bed

D) Any of the above

18. How should the shock be delivered in defibrillation?

A) Press and hold the shock button until the shock is delivered

B) Press and release the shock button and wait for the shock to be delivered

C) Press the shock button twice and wait for the shock to be delivered

D) Any of the above

19. How should the rhythm be checked after defibrillation?

A) Look at the monitor screen and identify the rhythm

B) Feel for a pulse and confirm the rhythm

C) Listen to the device's voice prompts and follow the instructions

D) A and B only

20. How often should defibrillation be repeated if needed?

A) Every 2 minutes

B) Every 3 minutes

C) Every 5 minutes

D) Every 10 minutes

**Attitude Questionnaire**

I have created a questionnaire to identify your attitude and barriers towards defibrillation, which is the delivery of an electric shock to the heart to restore its normal rhythm in cases of cardiac arrest. Please read each statement carefully and indicate how much you agree or disagree with it on a scale of 1 to 5, where 1 means strongly disagree and 5 means strongly agree. Please mark your answer by circling the number that corresponds to your choice.

1. I feel confident in using a defibrillator in the emergency department.

1 2 3 4 5

2. I think defibrillation is an effective and safe intervention for cardiac arrest.

1 2 3 4 5

3. I have sufficient knowledge and skills in using a defibrillator in the emergency department.

1 2 3 4 5

4. I have received adequate training and education on defibrillation in the emergency department.

1 2 3 4 5

5. I have access to a defibrillator with full monitor in the emergency department.

1 2 3 4 5

6. I have enough time and opportunity to practice using a defibrillator in the emergency department.

1 2 3 4 5

7. I have support and feedback from my colleagues and supervisors on defibrillation in the emergency department.

1 2 3 4 5

8. I am afraid of causing harm or injury to the patient or myself by using a defibrillator in the emergency department.

1 2 3 4 5

9. I am worried about legal or ethical issues related to defibrillation in the emergency department.

1 2 3 4 5

10. I face technical or logistical problems when using a defibrillator in the emergency department.

1 2 3 4 5

**Skills Questionnaire**

This questionnaire is designed to measure the skills in using a defibrillator with full monitor, which is a device that can deliver an electric shock to the heart to restore its normal rhythm in cases of cardiac arrest. You will be asked to perform a simulated scenario with a manikin and a defibrillator. You will be observed and scored by a trained evaluator using a checklist of 10 steps that you have to perform correctly. Each step will be scored as 1 point if performed correctly and 0 point if performed incorrectly or omitted. The total score will range from 0 to 10.

Scenario: You are working in the emergency department when a patient arrives with chest pain and shortness of breath. You suspect that he is having a heart attack and you attach him to a defibrillator with full monitor. You notice that his ECG shows ventricular fibrillation, which is a life-threatening arrhythmia that requires immediate defibrillation. You decide to perform defibrillation using manual mode.

**Steps:**

1. Turn on the device.

2. Select manual mode.

3. Attach electrodes to chest (right upper chest and left lower chest).

4. Check ECG.

5. Select energy level

6. Charge device.

7. Clear area (shout "clear" or "stand clear" or "I'm clear, you're clear, we're all clear").

8. Deliver shock (press shock button).

9. Check rhythm

10. Repeat steps 5-9 until rhythm changes or help arrives.
